# Educational attainment of adolescents treated in psychiatric inpatient care: a register study over 3 decades

**DOI:** 10.1007/s00787-022-02052-0

**Published:** 2022-08-06

**Authors:** Timo Holttinen, Nina Lindberg, Pekka Rissanen, Riittakerttu Kaltiala

**Affiliations:** 1 Department of Adolescent Psychiatry, Faculty of Medicine and Health Technology, Tampere University Hospital, Tampere University, Arvo Ylpön katu 34, 33014 Tampere, Finland; 2 Helsinki University and Helsinki University Hospital, Forensic Psychiatry, Helsinki, Finland; 3 Finnish Institute for Health and Welfare, Helsinki, Finland; 4 Vanha Vaasa Hospital, Vaasa, Finland

**Keywords:** Adolescent, Mental disorders, Education, Inpatient, Register study

## Abstract

Mental disorders may for various reasons impair educational attainment, and with far-reaching consequences given the impact of education on subsequent employment, social life, life choices and even health and mortality. This register-based study addresses trends in educational attainment among Finnish adolescents aged 13–17 with mental disorders severe enough to necessitate inpatient treatment between 1980 and 2010. Our subjects (*N* = 14,435), followed up until the end of 2014, were at greater risk of discontinuing education beyond compulsory comprehensive school or of lower educational attainment than their age-peers in general population. Only 50.0% had completed any post-comprehensive education compared to 84.9% in same-aged general population. Those at highest risk were males and those with organic, intellectual disabilities and developmental, externalizing disorders or schizophrenia group diagnoses. Despite improvements in adolescent psychiatric care, school welfare services and pedagogical support, risks have remained high. Greater effort in psychiatric treatment, school welfare and pedagogy are needed to combat this severe inequality.

**Supplementary Information:**

The online version contains supplementary material available at 10.1007/s00787-022-02052-0.

## Introduction

Adolescence is a crucial time for establishing personal values or ethical systems, adopting socially responsible behavior and making choices regarding one’s educational pathway [1]. Education affects subsequent employment, social life and even health and mortality [2–7]. Adolescents dropping out of secondary school are at markedly increased risk for sickness and disability in young adulthood [3] and are more likely to smoke or take drugs, to report suicide attempts in the preceding year and to have been arrested on suspicion for crimes [8]. Higher education is associated with healthier behavior and overall health [2, 5, 9]. Formal education has an ever-increasing role in working life [10, 11] accompanied by pressure to shorten the time needed to qualify and enter working life [12]. These developments may exacerbate the negative impact of adolescent mental disorders on ultimate educational level and position in working life.

### The Finnish education system

**Fig. 1:**
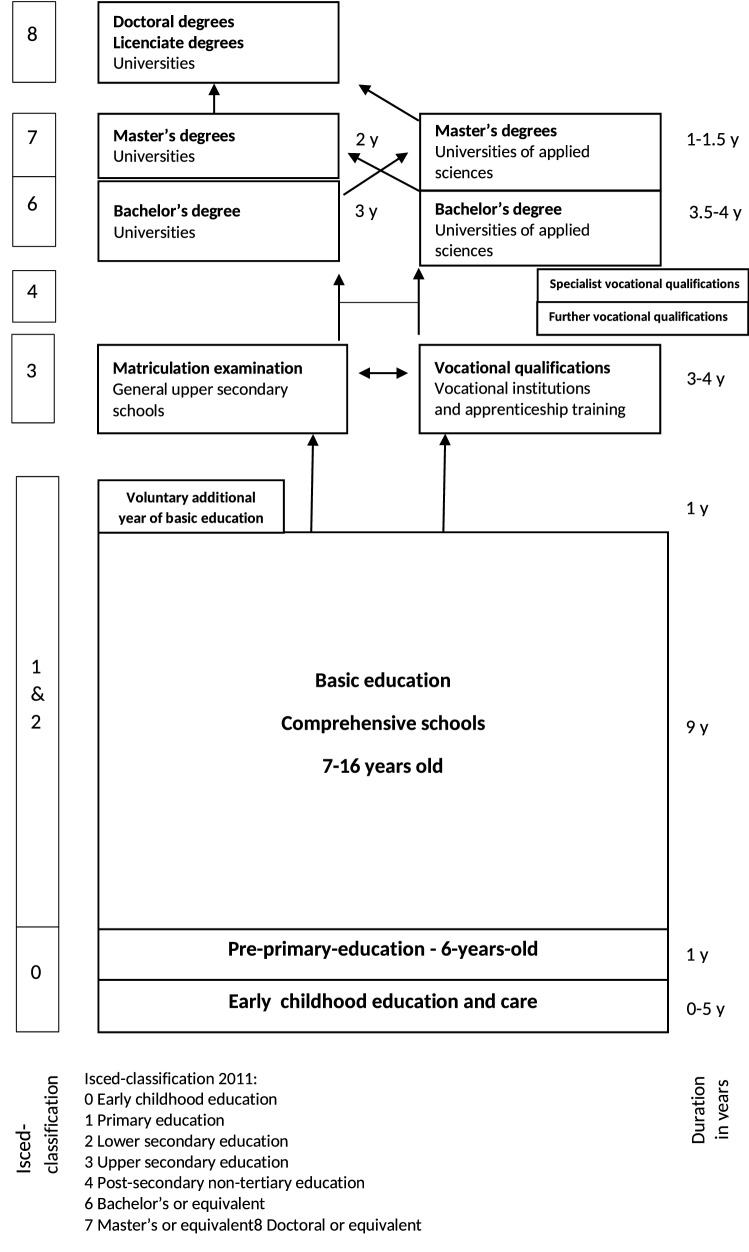
Education system in Finland

Education in Finland is publicly funded up to and including tertiary education and thus not contingent upon students’ socioeconomic status, ethnicity, or other considerations. Since the 1970s, education has been compulsory for 9 years of comprehensive school or until age 16. In 2021, the school-leaving age was raised to 18 years and compulsory education extended to include upper secondary education. Post-comprehensive education includes upper secondary education and tertiary education (Fig. 1).

Over 90–95% of the age cohort continue to upper secondary education [13]; thus, the educational level in Finland has risen steadily. In 1980, only 40.4% of those over 20 had completed at least upper secondary education and only 14.1% tertiary-level education, whereas in 2010, this reached 71.2% and 30.0% (1990: 53.3%/19.4%; 2000: 63.2%/25.3%) [14].

### Mental disorders in adolescence and education

Mental disorders may for various reasons impair educational attainment. In externalizing disorders (such as substance use disorders, conduct disorders and ADHD), truancy, lack of motivation and attention deficits [15–17] may affect school attendance and dropping out of school. In internalizing disorders (such as depression and anxiety disorders), fatigue, lack of interest, and cognitive symptoms (difficulties in concentration, learning, memory and problems attending school) may seriously impair learning and adjustment to school [18]. In psychotic disorders, both positive and negative symptoms may impair learning and adjustment to school through impairment of cognitive and social functioning [19–21]

The association between externalizing disorders and negative educational outcomes such as school dropout and lower educational attainment is well established [15–17]. The findings on internalizing disorders have been less consistent. In some studies, internalizing disorders have been associated with underachievement at school, problems with school attendance and failure to complete school education [22–27]. However, in other studies, no association has been found or this has diminished after controlling for confounding factors like parents and family SES or externalizing symptoms [19, 28, 29].

Positive developments in the availability and variety of adolescent psychiatric services in Finland since the 1980s [30] may also have helped adolescents with mental disorders to achieve higher educational goals. For example, neuropsychological assessments and rehabilitation have been more readily available since the 1980s [31], likewise more easily obtainable medication for depression and especially for ADHD. Efforts have moreover been made since the 1970s to improve access to student welfare services in schools and these were accelerated in the 2000s [32]. The range of special education for those with special needs has increased, resulting in the highest number of pupils receiving part-time special education in the mid-2000s. Support has evolved from separate special education into more part-time support for learning and school attendance [33]. Despite the emphasis on outpatient care, the need for adolescent psychiatric inpatient care appears to have increased between the late 1990s and 2010. However, mean length of inpatient stay has simultaneously decreased from 66 days (median 28) in the 1980s to 36 days (median 15) in the 2000s [34]. Inpatient care is only warranted when patients’ psychiatric symptoms are so severe (for example suicidality, psychosis, aggression) that outpatient services (including child protection services) do not suffice to ensure the safety of patients or others.

To summarize, increased and improved adolescent psychiatric services and school welfare services might have been expected to have resulted in better academic achievement despite psychiatric illness during adolescence. The policy to shorten the time spent in education and to transfer young adults earlier to working life may have counteracted the positive developments in services to help those with mental disorders. Need for inpatient treatment can be seen as an indicator of the severity of mental disorders. This register-based study aims to evaluate trends in educational attainment among adolescents aged 13 to 17 with mental disorders severe enough to warrant inpatient treatment for the first time between 1980 and 2010. Including study population from three different decades makes it possible observe the effects of the development in adolescent psychiatric services and school support systems. More specifically, we sought answers to the following questions.

1. Are psychiatric disorders requiring psychiatric inpatient care in adolescence differentially associated with lower educational attainment in later life?
2. What are the risk factors for not acquiring education beyond compulsory comprehensive school among those admitted to psychiatric hospital during adolescence?
3. Are there differences in educational attainment between adolescents hospitalized due to psychiatric disorders in different decades?

## Methods

This is a register-based follow-up study, the study population comprising all Finnish citizens admitted between 1980 and 2010 for their first ever psychiatric inpatient treatment at age 13–17 in Finland. Information on inpatient care in psychiatric hospital was obtained from the Patient Discharge Register (used between 1969 and 1993) and the Care Register for Health Care (from 1994). Altogether 17,112 adolescents aged 13–17 were admitted to psychiatric inpatient care for the first time (in their lives) in 1980–2010. The subjects were followed up in registers until 2014.

The study data contains diagnostic information from three versions of the International Classification of Diseases (ICD). The WHO conversion tables were used to recode ICD-8 and ICD-9 diagnoses as ICD-10 diagnoses [35]. In the analyses, primary diagnoses according to ICD-10 were classified into organic, intellectual disability and developmental (diagnostic groups F00–09, F70–79, F80–89, G-diagnoses), Schizophrenia group (F20–29), mood disorders (F30–39) anxiety disorders (F40–48), behavioral syndromes associated with physiological disturbances and physical factors (F50–59), externalizing disorder (F10–19, F60–69, F90–92), emotional disorders of childhood (F93–99) and social reasons (Z-codes). Age at index admission was classified to early (13–14 years) and middle (15–17 years) adolescence.

Information on highest attained post-comprehensive school education was obtained from the Statistics Finland Register of Completed Education and Degrees. The statistics include all upper secondary and tertiary education options with harmonized concepts and classifications. In the analyses, highest education obtained was classified as comprehensive school only, upper secondary education (general upper secondary school and vocational qualifications and tertiary education (university of applied sciences, university). Data on enrollment in post-comprehensive school studies in 2014 were obtained from the Statistics Finland Progress of Studies register.

For the present study, we included those aged 20–49 years who were alive and who had received psychiatric diagnoses or organic, neurological (G-group), intellectual disability or developmental diagnosis or social reasons (Z-code) diagnosis as the primary diagnosis at index admission. On 1.3% of these (197/14,632; demographic information in Supplementary Table 1), no education information was available in 2014 and they were excluded from the study. The lower age limit was chosen assuming that the vast majority would have completed upper secondary education by age 20 in Finland [12, 36]. This upper age limit was chosen to ensure equal age groups. Because subjects had different follow-up times and, therefore, different amounts of time to attain education, the study population was categorized into age groups based on subjects’ age at the end of 2014. These age groups were 20–29, 30–39 and 40–49 years. To compare the educational attainment of the study population to that in sex- and age-matched general population, information on the educational attainment of Finnish population was gathered from the Official Statistics of Finland: Educational Structure of Population [14].

### Statistical analyses

Cross-tabulations with Chi-square statistics with effect sizes were used to investigate associations between sex, age and primary diagnosis at first inpatient care and a subject’s attainment of a university degree or pursuit of post-comprehensive school education at the end of 2014. The highest educational levels attained were compared between former patients and general population in sex and age groups using cross-tabulations and Chi-square statistics.

Logistic regression analysis was used to assess the odds ratios for no post-comprehensive school education (attained or pursued in 2014). Sex, categorized age at index admission and psychiatric primary diagnosis at index admission were used as covariates. Sex and primary diagnosis interactions were also examined to ascertain if sex moderated the association between diagnosis and education attainment using sex as a moderator. Logistic regression analyses were performed separately for each age group as psychiatric treatment and education have constantly developed over the years.

SPSS 27.0 version and MedCalc’s comparison of proportions calculator were used for statistical analyses. P-value 0.05 was used as a threshold for statistical significance.

## Results

Of the study population (*n* = 14,435), 38.8% (*n* = 5603) were males and 61.2% (*n* = 8832) females. Information on the age of the study population age at first admission to inpatient treatment and in 2014 and primary diagnoses in first psychiatric inpatient care are presented in Table 1.

**Table 1:** Age in first inpatient care and in 2014 and primary diagnosis in first psychiatric inpatient care % (*n*)

	Male (*N* 5603)	Female (*N* 8832)	*p* value (Cohen’s *h*)
Age at first inpatient care episode			
13–14 years	30.9% (1730)	27.0% (2389)	< 0.001 (0.09)
15–17 years	69.1% (3873)	73.0% (6443)	< 0.001 (0.09)
Age in 2014			
20–29 years	55.5% (3109)	65.7% (5803)	< 0.001 (0.21)
30–39 years	26.9% (1507)	23.5% (2075)	< 0.001 (0.08)
40–49 years	17.6% (987)	10.8% (954)	< 0.001 (0.2)
Primary diagnosis in first psychiatric inpatient care			
Organic, intellectual disability and developmental (F00–09, F70–79, F80–89, G-diagnoses)	4.8% (270)	1.6% (145)	< 0.001 (0.19)
Schizophrenia group (F20–29)	14.2% (793)	9.8% (869)	< 0.001 (0.14)
Mood disorders (F30–39)	19.8% (1110)	35.4% (3128)	< 0.001 (0.35)
Anxiety disorders (F40–48)	18.9% (1061)	18.6% (1647)	0.65 (0.01)
Behavioral syndromes associated with physiological disturbances and physical factors (F50–59)	1.0% (54)	8.0% (708)	< 0.001 (0.37)
Externalizing disorders (F10–19, F60–69, F90–92)	28.8% (1614)	16.2% (1428)	< 0.001 (0.3)
Emotional disorders of childhood (F93–99)	8.0% (448)	6.9% (608)	0.01 (0.04)
Social reasons (Z-codes)	4.5% (253)	3.4% (299)	< 0.001 (0.06)

*p values (Cohen’s *h* effect sizes) for categorical proportion differences between sexes

### Education attained

**Fig. 2:**
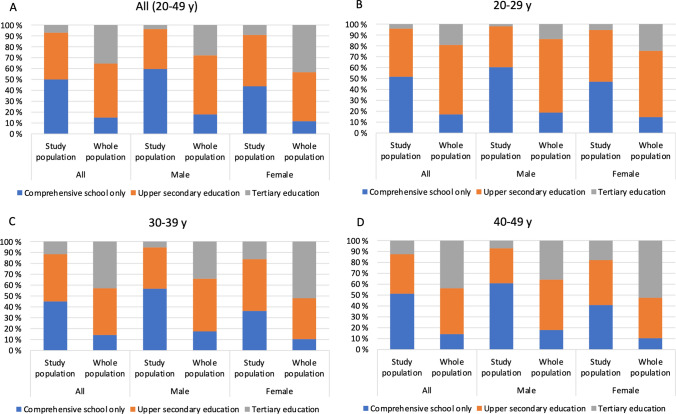
Highest school education completed in study population in 2014 compared to that of whole population by age group

As Fig. 2A shows, 50.0% of the study population had no post-comprehensive school education, 42.9% had completed upper secondary education and 7.1% had completed tertiary education in 2014. In general population, 84.9% of Finns aged 20–49 had completed at least some kind of post-comprehensive school education (49.7% upper secondary education and 35.2% tertiary education). In every age group (age in 2014) and among both males and females, the proportion of those with no post-comprehensive school education was much higher in the study population than in general population and was highest in the youngest age group (Fig. 2B). In addition, highest educational level attained was lower in every age group in the study population. More precise numerical information is presented in Supplementary Table 2.

Of those with comprehensive school education only (*n* = 7224), 25.6% were enrolled in upper secondary or tertiary education in 2014, females more often than males (30.9% (1200/3881) vs. 19.4% (649/3343), *p* < 0.00, phi 0.131) Being enrolled in post-comprehensive school education was more common in younger age groups (age 20–29: 35.8% (1654/4622), 30–39: 10.4% (168/1608), 40–49: 2.7% (27/994), *p* < 0.001, Cramer’s *V* 0.315). No data were available on the whole population.

### Risk factors for not achieving post-comprehensive school education

Altogether 37.2% of the study population had not completed or were not enrolled in post-comprehensive school education in 2014. As seen in Table 2, this was more likely among males (48.1% (2694/5603) vs. 30.4% (2681/8832) in females (*p* < 0.001, phi 0.13) and in older age groups. In females, those entering psychiatric inpatient care for the first time in early adolescence (13–14 years) were slightly more likely to have no post-comprehensive school education or not to be enrolled in it than those first admitted during middle adolescence (15–17 years). In males, age at first admission to psychiatric inpatient treatment was not associated with subsequent educational attainment.

**Table 2:** No post-comprehensive education completed or pursued in year 2014, by age and primary diagnosis at index admission and age in 2014 (% (*n*/*N*))

	Males	Females	All
Age at first inpatient care			
13–14 years	46.7% (808/1730)	32.1% (768/2389)	38.3% (1576/4119)
15–17 years	48.7% (1886/3873)	29.7% (1913/6443)	38.8% (3799/10316)
*p* (Cramer’s *V* effect size) *for categorical proportion difference of age groups in first inpatient care	0.168 (0.018)	0.026 (0.024)	0.107 (0.013)
Age in 2014			
20–29 years	42.6% (1325/3109)	28.3% (1643/5803)	33.3% (2968/8912)
30–39 years	52.0% (784/1507)	31.6% (656/2075)	40.2% (1440/3582)
40–49 years	59.3% (585/987)	40.0% (382/954)	49.8% (967/1941)
*p* (Cramer’s *V* effect sizes) *for categorical proportion differences of age groups in 2014	< 0.001 (0.131)	< 0.001 (0.079)	< 0.001 (0.119)
Primary diagnosis in first inpatient care			
Organic, intellectual disability and developmental (F00–09, F70–79, F80–89, G-diagnoses)	54.4% (147/270)	46.9% (68/145)	51.8% (215/415)
Schizophrenia group (F20–29)	49.1% (389/793)	38.1% (331/869)	43.3% (720/1662)
Mood disorders (F30–39)	39.2% (435/1110)	25.4% (793/3128)	29.0% (1228/4238)
Anxiety disorders (F40–48)	47.5% (504/1061)	30.1% (496/1647)	36.9% (1000/2708)
Behavioral syndromes associated with physiological disturbances and physical factors (F50–59)	22.2% (12/54)	11.9% (84/708)	12.6% (96/762)
Externalizing disorders (F10–19, F60–69, F90–92)	55.3% (893/1614)	39.6% (565/1428)	47.9% (1458/3042)
Emotional disorders of childhood (F93–99)	44.2% (198/448)	36.5% (222/608)	39.8% (420/1056)
Social reasons (Z-codes)	45.8% (116/253)	40.8% (122/299)	43.1% (238/552)
*p* (Cramer’s *V* effect sizes) *for categorical proportion differences of diagnosis groups	< 0.001 (0.128)	< 0.001 (0.178)	< 0.001 (0.194)

The former patients with primary diagnoses of behavioral syndromes (F50–59) were the only patient group where the proportion of those with no education beyond comprehensive school was lower than in general population (Fig. 2A and Table 2).

In logistic regression analyses (Table 3), male sex was a risk for no further education in each age group. There was no significant difference according to age at index admission. All other diagnostic groups were at significantly higher risk than those with behavioral syndromes group (F50–59) diagnoses as the primary diagnosis at index admission. Those with organic, intellectual disability and developmental (F00–09, F70–79, F80–89) diagnoses, externalizing disorders (F10–19, F60–69, F90–92) or schizophrenia group diagnoses (F20–29) were at highest risk. In addition, those with internalizing disorder diagnoses (mood disorders F30–39 or anxiety disorders F40–48) were at higher risk than those with behavioral syndromes group (F50–59) diagnoses.

**Table 3:** Odds ratios (95% confidence intervals) for no post-comprehensive education completed or pursued in 2014 (*N* = 14,435)

	20–29 years (*n* = 8912)		30–39 years (*n* = 3582)		40–49 years (*n* = 1941)	
	OR (95% CI)	*p*	OR (95% CI)	*p*	OR (95% CI)	*p*
Sex						
Female	Ref		Ref		Ref	
Male	1.6 (1.4–1.7)	< 0.001	2.1 (1.8–2.4)	< 0.001	2.0 (1.7–2.4)	< 0.001
Age at index admission						
13–14 years	Ref		Ref			
15–17 years	1.0 (0.9–1.1)	0.760	0.9 (0.8–1.0)	0.111	1.1 (0.9–1.4)	0.312
Primary diagnosis in index admission^a^						
Behavioral syndromes associated with physiological disturbances and physical factors (F50–59)	Ref		Ref		Ref	
Organic, intellectual disability and developmental (F00–09, F70–79, F80–89, G-diagnoses)	5.0 (3.5–7.2)	< 0.001	5.2 (2.7–10.3)	< 0.001	7.4 (3.1–17.5)	< 0.001
Externalizing disorders (F10–19, F60–69, F90–92)	4.9 (3.7–6.4)	< 0.001	4.5 (2.9–7.2)	< 0.001	6.7 (3.0–14.6)	< 0.001
Schizophrenia group (F20–29)	3.9 (2.9–5.3)	< 0.001	3.4 (2.1–5.4)	< 0.001	5.3 (2.4–11.4)	< 0.001
Mood disorders (F30–39)	2.5 (1.9–3.3)	< 0.001	2.6 (1.7–4.1)	< 0.001	3.2 (1.4–7.1)	0.006
Anxiety disorders (F40–48)	2.9 (2.2–3.9)	< 0.001	3.1 (2.0–5.0)	< 0.001	4.7 (2.2–10.2)	< 0.001
Emotional disorders of childhood (F93–99)	3.9 (2.9–5.4)	< 0.001	3.3 (1.0–5.4)	< 0.001	3.7 (1.6–8.8)	0.003
Social reasons (Z-codes)	3.9 (2.9–5.4)	< 0.001	4.6 (2.7–7.9)	< 0.001	3.6 (1.6–8.5)	0.003

^a^Sex was not a statistically significant moderator of associations between diagnosis and educational attainment (*p* values 20–29 years group: 0.08, 30–39 years: 0.36, 40–49 years: 0.06)

## Discussion

Highest educational attainment among former adolescent psychiatric inpatients across age groups was considerably lower than among Finnish general population. The study population included an especially high proportion of those who had only completed comprehensive school education, with a significantly large difference from general population, especially in males. Except for behavioral syndromes group (F50–59), every diagnostic group had a two- to threefold greater proportion of those who had completed only comprehensive school education when compared to general population. Especially in males, those with externalizing disorder (F10–19, F60–79, F90–92) diagnoses differed from those with other diagnoses. These findings are more noticeable when compared to studies on general psychiatric problems or general population [18, 19, 37–49]. While mental health problems predispose to lower educational attainment, those with psychiatric symptoms severe enough to require inpatient care are at greater risk. The failure of former adolescent psychiatric inpatients to attain educational levels comparable to same-aged general population could be related to the nature of the disorders themselves or to the stigma related to psychiatric morbidity, but may also signify that, despite increases in the availability and variety of adolescent psychiatric services and welfare services in school and special education and other support for learning, society has failed to adequately promote the integration of adolescents with mental disorders into education, with long-term negative implications for their integration into working life.

With the exception of those with organic, intellectual disability and developmental group diagnoses (F00–09, F70–79, F80–89, G-diagnoses), those with externalizing disorders were at highest risk of having no post-comprehensive school education. This concurs with the findings of earlier studies [15, 16, 39] and may reflect the impact of neurocognitive and motivational deficits associated with externalizing disorders [16, 40, 41]. These deficits may also characterize those whose index admissions were due to diagnoses in the emotional disorders of childhood group (F93–99) and the social reasons group (z-codes) as these diagnoses are often set in the absence of other obvious psychiatric problems. The risk persisted in the youngest age groups even though the pharmacological treatment of attention deficits and hyperactivity, for example, improved vastly during the 2000s [42].

In addition to the well-known impairments caused by cognitive deficits found, for example, in schizophrenia group (F20–29) diagnoses, impaired social functioning, seen in many mental disorders, may also have a negative effect on the skills needed for education [21, 43–45]. Adolescents with anxiety disorders (F40–48), especially social phobia, face many academic and social difficulties and experience higher levels of peer victimization, which may lead to refusal to attend school, slow academic progress or failure to enter higher education [46, 47]. Adolescence is a stage of life associated with the rapid acquisition of new information and making far-reaching choices, for example, in education. Mood disorders (F30–39) are often associated with cognitive dysfunctions persisting beyond symptom recovery [48, 49], and this may have particularly damaging and long-lasting effects on education. It has been proposed that the impact of mood disorders on educational attainment may be to delay, rather than to prevent, completion of higher education [24]. However, in this study, the risk of exclusion from higher education was present in every age group. In this case, lower educational attainment compared with same-aged general population might well no longer be seen in the oldest age groups, which was not the case in the present study.

In addition to the negative effect of the symptoms, mental disorders may also have a negative impact on educational attainment due to the accompanying stigma. Stigma may reduce willingness to seek help and impair treatment adherence, undermine treatment outcome, impair well-being and, especially during adolescence, influence personal identity and independence in the long term [50–53]. Adolescents with mental disorders are deemed more stigmatized than peers with other health problems [52]. Stigma may manifest in general devaluation, bullying, being underestimated by others or outright social rejection, which may impair adolescents’ willingness to pursue education [50]. In addition, adolescents with mental disorders may be branded as having a disability, which may lead to lower educational expectations on the part of parents and teachers. For example, Schifrer [54] found that among similarly achieving and behaving adolescents the odds of teachers expecting a bachelor’s degree education or higher for adolescents labeled as having a learning disability were 82% lower than for adolescents without this and that adolescents’ educational expectations are partially mechanized through parents’ and particularly teachers’ lower expectations. Therefore, while learning difficulties associated with many mental disorders may require support from special education services, those services may, while improving students’ opportunities to learn, engender stigma.

This study represents 3 decades of development of adolescents’ mental health services and the education system. Subjects aged 40–49 years in 2014 were in adolescent psychiatric inpatient care mainly in the 1980s, those aged 30–39 in the 1990s and those aged 20–29 in the 2000s. In spite of considerable improvement in support services for those in need in both education and mental health services [13, 30, 33] those adolescents with psychiatric disorders requiring psychiatric inpatient treatment in the 2000s were nevertheless at similar risk for not pursuing higher education to those in inpatient care in the 1980s. This may be because psychiatric disorders impair academic performance so profoundly that even if support is arranged at school, it does not suffice to alleviate the effects of psychiatric symptoms. Another possibility is that the focus on adolescents’ social environments and skills and the kind of support needed is still inadequate. Third, stigma related to mental disorders may hinder functioning at school and the readiness to offer and accept support. Systematically lowered educational attainment across diagnostic categories and across decades is not only a matter of persistent inequality. Given the importance of education today, lack of post-comprehensive school education may impair employment prospects, lead to lower socioeconomic status and so to poorer physical and mental health [3, 4, 55].

### Methodological considerations

The strength of this study is the large nationwide data covering a long study period of 3 decades. The data suffer from no distorting effect of regional differences in adolescent mental health services or educational opportunities. The Finnish national register data used in this study are of high quality and collected directly and mandatorily from healthcare and education authorities. The Finnish national registers make it possible to study large patient groups and to collate information on different registers on an individual level using the unique personal identity number assigned to each permanent resident in Finland.

A first limitation is the risk of inaccuracies in both diagnostic and education data. Diagnoses were supplied by clinicians and may, therefore, contain clinician-related bias. For example, inpatients with ADHD diagnoses are often treated for associated conduct problems although no conduct disorder has been diagnosed. However, diagnostic practices have been shown to be reliable in Finnish psychiatric inpatient care [56] and as we analyzed the data on the level of diagnostic main classes, the risk of individual clinician-related bias is reduced. As this study focused solely on primary diagnoses, the effects of comorbid psychiatric symptoms (e.g., intellectual disabilities) were not explored. However, as inpatient care usually focuses on the main psychiatric symptoms, the primary diagnosis is the best indicator of patients’ problems and is the only diagnosis invariably recorded in registers. In addition, services required by most of those with intellectual disabilities are provided by separate intellectual disability services. As this study focused on adolescents aged 13–17, some diagnostic phenotypes might not have been fully apparent, causing misinterpretation of subsequent psychiatric disorders. Education completed abroad may not be comprehensively covered in the registers studied. However, Statistics Finland actively supplements data on education, for example through information exchange with other countries, and on the other hand, there is no reason to assume that former patients would have more commonly completed education abroad than the same-aged general population.

A second limitation is that register-based data only enable the observation of rough trends in educational attainment. A more detailed analysis of the impact of psychiatric symptoms or neurocognitive factors on school performance would entail examining individual patient records. In addition, the study subjects’ parents’ educational status was unfortunately not known in the present study. In some studies, parental socioeconomic status has been thought to have a greater impact than psychiatric disorders on offspring’s attained education [16, 57], while in some studies, the impact has not been so clear [20, 23, 58]. However, as the social reasons diagnosis group (Z-code) contains diagnoses of socioeconomic factors influencing health, and in this study, those with social reasons diagnoses (Z-code) as primary diagnoses at index admission were at higher risk for no further education than those with mood disorder diagnoses, it is possible that socioeconomic factors also affect other diagnostic groups.

It should moreover be noted that psychiatric disorders warranting inpatient care likely represent the most severe end of the continuum of psychiatric morbidity. Conditions only requiring care in the community may have less impact on educational attainment.

## Conclusion

Adolescents with psychiatric disorders requiring inpatient care are at considerable risk for not continuing their education beyond compulsory comprehensive school or of attaining lower educational levels than their age-peers in general population. This has not changed over time despite improvements in adolescent psychiatric care, school welfare services and pedagogical support. The risk for low educational attainment among former adolescent psychiatric inpatients is particularly pronounced among males. Greater effort in psychiatric treatment, school welfare and pedagogical effort are needed to tackle this severe inequality. Psychiatric symptoms, cognition, social integration and stigma all need to be considered.

### Supplementary Information

Below is the link to the electronic supplementary material.

Supplementary file1 (PDF 103 kb)

Supplementary file2 (PDF 71 kb)

## Data Availability

Availability of the datasets used and/or analyzed in this study is subject to data permits from the appropriate Finnish register authorities. More information from the corresponding author upon request.
