# Supplementary material for: Educational attainment of adolescents treated in psychiatric inpatient care: a register study over 3 decades
Source: Eur Child Adolesc Psychiatry. 2022 Aug 6;32(11):2163–73. doi: 10.1007/s00787-022-02052-0 (PMC10576713; doi:10.1007/s00787-022-02052-0)
Supplement: Supplementary file 1 — Supplementary file1 (PDF 103 kb) [file 787_2022_2052_MOESM1_ESM.pdf]

Supplementary Table 1:<br>No educational information available in 2014 (missing data) among those alive and 20-49 years old in 2014 and having psychiatric (F), neurological (G) or social reasons diagnosis in index admission	
**All**	1.3 % (197/14632)
**Sex:**	
Male	1.1 % (64/5667)
Female	1.5 % (133/8965)
p (phi) vs sexes	0.07 (0.02)
**Age at inpatient care:**	
13-14 yrs	1.3 % (53/4172)
15-17 yrs	1.4 % (144/10316)
p (phi) vs age groups	0.61 (0.004)
**Age in 2014:**	
20-29 yrs	0.9 % (83/8995)
30-39 yrs	2.0 % (72/3582)
40-49 yrs	2.1 % (42/1983)
p (Cramer's V) vs. age groups	<0.001 (0.05)
**Primary diagnosis in first inpatient care:**	
Organic, intellectual disability and developmental (F00-09, F70-79, F80-89, G-diagnoses)	1.2 % (5/420)
Schizophrenia group (F20-29)	0.9 % (15/1677)
Mood disorders (F30-39)	1.3 % (55/4293)
Anxiety disorders (F40-48)	1.5 % (42/2708)
Behavioural syndromes associated with physiological disturbances and physical factors	2.6 % (20/782)
Externalizing disorders (F10-19, F60-69, F90-92)	1.3 % (40/3082)
Emotional disorders of childhood (F93-99)	0.9 % (10/1066)
Social reasons (Z-codes)	1.8 % (10/562)
p (Cramer's V) vs diagnosis groups	<0.05 (0.03)
