# Supplementary material for: Educational attainment of adolescents treated in psychiatric inpatient care: a register study over 3 decades
Source: Eur Child Adolesc Psychiatry. 2022 Aug 6;32(11):2163–73. doi: 10.1007/s00787-022-02052-0 (PMC10576713; doi:10.1007/s00787-022-02052-0)
Supplement: Supplementary file 2 — Supplementary file2 (PDF 71 kb) [file 787_2022_2052_MOESM2_ESM.pdf]

Supplementary table 2: Highest school education completed in study population compared to that of whole population by age groups , % (n) (with p-values and Cohen's h effect sizes)

	**20-29 y**			**30-39 y**			**40-49 y**			**All (20-49y)**		
**Males**	Study population (N 3109)	Whole population (N 348782)	p-values (h)	Study population (N 1507)	Whole population (N 360229)	p-values (h)	Study population (N 987)	Whole population (N 342288)	p-values (h)	Study population (5603)	Whole population (1051299)	p-values (h)
Comprehensive school only	60.6 % (1883)	19.0 % (66346)	<0.001 (0.89)	56.9 % (857)	17.6 % (63492)	<0.001 (0.84)	61.1 % (603)	17.9 % (61314)	<0.001 (0.92)	59.7 % (3343)	18.2 % (191152)	<0.001 (0.8)
Upper secondary education	37.4 % (1162)	67.4 % (235069)	<0.001 (0.61)	38.0 % (572)	48.2 % (173659)	<0.001 (0.21)	32.1 % (317)	46.5 % (159285)	<0.001 (0.3)	36.6 % (2051)	54.0 % (568013)	<0.001 (0.35)
Tertiary education	2.1 % (64)	13.6 % (47367)	<0.001 (0.46)	5.2 % (78)	34.2 % (123078)	<0.001 (0.79)	6.8 % (67)	35.6 % (121689)	<0.001 (0.75)	7.1 % (209)	35.2 % (292134)	<0.001 (0.73)
**Females**	Study population (N 5803)	Whole population (N 332869)	p-values (h)	Study population (N 2075)	Whole population (N 339857)	p-values (h)	Study population (N 954)	Whole population (N 331708)	p-values (h)	Study population (8832))	Whole population (1004164)	p-values (h)
Comprehensive school only	47.2 % (2739)	14.9 % (49470)	<0.001 (0.72)	36.2 % (751)	10.4 % (35320)	<0.001 (0.63)	41.0 % (391)	10.4 % (34644)	<0.001 (0.73)	43.9 % (3881)	11.9 % (119434)	<0.001 (0.74)
Upper secondary education	47.5 % (2755)	60.8 % (202432)	<0.001 (0.27)	47.8 % (991)	37.5 % (127194)	<0.001 (0.21)	41.1 % (392)	37.2 % (123246)	0.013 (0.08)	46.9 % (4138)	45.1 % (452872)	<0.001 (0.04)
Tertiary education	5.3 % (309)	24.3 % (80967)	<0.001 (0.57)	16.0 % (333)	52.1 % (177073)	<0.001 (0.79)	17.9 % (171)	52.4 % (173818)	<0.001 (0.75)	9.2 % (813)	43.0 % (431858)	<0.001 (0.81)
**All**	Study population (N 8912)	Whole population (N 681651)	p-values (h)	Study population (N 3582)	Whole population (N 699816)	p-values (h)	Study population (N 1941)	Whole population (N 673996)	p-values (h)	Study population (14435)	Whole population (2055463)	p-values((h)
Comprehensive school only	51.9 % (4622)	17.0 % (115816)	<0.001 (0.76)	44.9 % (1608)	14.1 % (98812)	<0.001 (0.7)	51.2 % (994)	14.2 % (34644)	<0.001 (0.82)	50.0 % (7224)	15.1 % (310586)	<0.001 (0.77)
Upper secondary education	43.9 % (3917)	64.2 % (437501)	<0.001 (0.41)	43.6 % (1563)	43.0 % (300853)	0.47 (0.01)	36.5 % (709)	41.9 % (123246)	<0.001 (0.11)	42.9 % (6189)	49.7 % (1020885)	<0.001 (0.14)
Tertiary education	4.2 % (373)	18.8 % (128334)	<0.001 (0.48)	11.5 % (411)	42.9 % (300151)	<0.001 (0.74)	12.3 % (238)	43.8 % (173818)	<0.001 (0.73)	7.1 % (1022)	35.2 % (723992)	<0.001 (0.73)
